# Regional Sustainable Development Analysis Based on Information Entropy—Sichuan Province as an Example

**DOI:** 10.3390/ijerph14101219

**Published:** 2017-10-13

**Authors:** Xuedong Liang, Dongyang Si, Xinli Zhang

**Affiliations:** 1 School of Economics, Sichuan University, Chengdu 610065, China; liangxuedong@scu.edu.cn; 2 The Economy and Enterprise Development Institute, Sichuan University, Chengdu 610065, China; 2016225025033@stu.scu.edu.cn

**Keywords:** regional sustainable development capacity, information entropy, Brusselator model, capability measurement, evaluation index system

## Abstract

According to the implementation of a scientific development perspective, sustainable development needs to consider regional development, economic and social development, and the harmonious development of society and nature, but regional sustainable development is often difficult to quantify. Through an analysis of the structure and functions of a regional system, this paper establishes an evaluation index system, which includes an economic subsystem, an ecological environmental subsystem and a social subsystem, to study regional sustainable development capacity. A sustainable development capacity measure model for Sichuan Province was established by applying the information entropy calculation principle and the Brusselator principle. Each subsystem and entropy change in a calendar year in Sichuan Province were analyzed to evaluate Sichuan Province’s sustainable development capacity. It was found that the established model could effectively show actual changes in sustainable development levels through the entropy change reaction system, at the same time this model could clearly demonstrate how those forty-six indicators from the three subsystems impact on the regional sustainable development, which could make up for the lack of sustainable development research.

## 1. Introduction

Along with the world’s economic structural adjustment, China’s economic development pressure has been gradually increasing, with regional development being often unbalanced, uncoordinated, and discontinuous. As a large developing country, China has a large population, resource shortages, ecological imbalances, environmental pollution, and serious social and environmental problems such as rising unemployment [1]. Therefore, only by insisting on sustainable development strategies in China to implement economic, social, and environmentally coordinated development strategies can the present problems be fundamentally solved. As the basic requirement for the implementation of a scientific development perspective, sustainable development needs to consider all aspects of economic construction, political construction, cultural construction, social construction and ecological civilization construction, and needs to consider regional development, economic and social development, and the harmonious development of society and nature.

Since 2007, more than half of the world’s population has been living in cities. Due to the acceleration of the urbanization process and the deterioration of the organization and service functions of urban ecosystems, economic activities, ecological environmental capacity, and even social sustainable development have sharply reduced [2,3]. In 1992 the Chinese government introduced for the first time a sustainable development strategy into the long-term planning of economic and social development. However, after more than 20 years’ development, there are still many issues in the sustainable development of Chinese regions, while at the same time the regional sustainable development problems have been found to be increasingly complex, such as concentration of the population, traffic jams, housing shortages, resource shortages, biodiversity reductions, “heat island” effects, noise, and air and water pollution. Regional sustainable development emphasizes the coordinated development of the economy, society and the ecology to promote the regional economy, improve residents' lives, ensure an efficient use of resources and maintain the environment [4,5]. Because of the complex interrelationships and interactions between the society and the environment, regional sustainable development research has become increasingly popular. Significant research has focused on regional sustainable development and its influencing factors by considering sustainable development path selection and capacity improvement problems with a view to change the traditional regional development paths to develop new paths for regional sustainable development.

Berry and Portney examined 50 large U.S. cities using two original databases as the foundation and explored the actual behavior of the cities with respect to sustainability and economic development policies. The results showed that the high number of programs aimed at achieving sustainability were linked to the inclusion of environmental advocacy groups. This relationship did not appear to compromise business advocacy groups and the inclusion of environmental groups in the policymaking was supported by the high rates of economic growth within the cities [6]. Fang et al. examined the sustainable development of an innovative city as the research object and argued that this necessary transition to sustainable development was being constrained by a series of bottlenecks in investment, income, techniques, contributions, and talents [7]. Zhang et al. studied the effects of infrastructure development, land use change, industrial structure and income on sustainable urban development. The results suggested that moderate land use change with steady economic growth was the key to sustainable urban development [8]. Through extensive investigation and research, Tweed and Sutherland focused on the link between sustainable urban development and cultural heritage and found that building a culture within a city could play an important role in sustainable urban development [9].

Significant research has examined regional sustainable development from an application perspective by establishing evaluation systems and evaluation models which analyzed the ecological environment, economic development and social construction, providing a reference for the promotion of regional sustainable development capacity. With the emergence of a series of ecological problems, Zhao et al. introduced a methodology for urban eco-security evaluation and applied a fuzzy comprehensive evaluation (FCE) calculation procedure. Statistical data during the period of 2005–2012 from Mianyang was examined using this methodology, which found that critical security and slight insecurity had a main impact on the urban ecological environment [10]. However, such indices are difficult to measure and quantify without sufficient spatial-temporal analysis. Shen et al. established an integrated model based on hierarchical index system for monitoring and evaluating urban sustainability. This was able to provide a theoretical basis for a comprehensive assessment of urban sustainability from a spatial-temporal perspective [11]. Yin et al. used eco-efficiency as an index to measure urban sustainable development and applied a data envelopment analysis model to describe the eco-efficiency of 30 Chinese provincial capital cities. The results showed that almost half the cities were fairly eco-efficient [12]. Javadian et al., focusing on the environmental dimension of sustainable urban development, conducted an environmental suitability analysis of educational land use in Tehran using AHP and GIS. By using the two models they were better able to determine which locations were environmentally suitable for educational land use, thereby providing a reference for sustainable urban development [13].

From the literature review, it can be seen that significant work has been done in improving regional innovation environments, enhancing innovation capacities, and changing regional development ideas. However, with increased focus on sustainable development problems, the requirements for regional sustainable development capacity have been continually extended. This is because of the need to consider the “economy-society-nature” relationship within complex sustainable development systems and the many factors that must be considered. To effectively define the data to ensure useful information to assess regional sustainable development ability, it is important to adopt appropriate data mining methods to analyze the index data and the target data. In order to assess the regional sustainable development capacity measurement process, the concept of information entropy and dissipative structure theory are combined to allow for an analysis of the performance of a regional operation system entropy change from the perspective of entropy flow transformation, which uses regional economic development, social development and ecological development as the point of contact. Because the dissipative structure theory developed from complex system theory is well justified and positioned to capture the complex and dynamic nature of the regional sustainable systems, and the regional system organization, functions and mechanisms are well described, thus Information entropy and dissipative structure theory provide an appropriate reference for regional sustainable development capacity evaluation research. Besides, to effectively define the data to ensure useful information to assess regional sustainable development ability, it is important to adopt appropriate data mining methods to analyze the index data and the target data.

The main purpose of the research in this paper is summarized as follows: first, a regional sustainable development capacity evaluation index system is established based on the previous scholars about the index system of regional sustainable development research results. Then, the index system is divided according to certain properties, and the entropy change analyzed. Regional sustainable development capacity is then studied using the information entropy concept and dissipative structure theory.

## 2. Measurement Index System of Regional Sustainable Development Capacity

How to get the proper and objective measure and evaluation about sustainability is one of the most fundamental issues of sustainability research [14,15,16,17]. The index system used in this paper was from a database from a previous regional sustainable development capacity study. The index system included most of the data needed to measure regional sustainable development capacity and the potential impacts. Regional sustainable development capacity analysis needs to comprehensively consider the sustainable development of the economy, the ecological environment and society, so that the real regional sustainable development capacity is reflected.

Economic sustainable development capacity refers to the economic elements of the regional sustainable development capacity study. On the one hand, economic development is the foundation of region social development, on the other hand, the economic growth at the cost of ignoring social development for the long run is unsustainable [18]. To comprehensively reflect the level of the regional economy, the regional economic sustainable development capacity needs to include all existing and potential economic capacity. Ecological environmental sustainable development capacity refers to the environmental elements of regional sustainable development capacity, which requires not only research on the regional environmental protection construction ability, but also on the coordination and development abilities of the regional environment and its resources. Social sustainable development capacity refers to the social factors of regional sustainable development capacity, which mainly focuses on the impact on the citizens’ life of the regional social mechanism and includes the effects of social progress as well as the effects of education and daily entertainment conditions [19]. 

Due to the multifaceted overlapping contents of the economy-environment-society complicated system’s components [20,21], the regional system is a multi-level, multi-functional and dynamic complex system which encompasses economic structure, social structure and natural structure. Each subsystem in this complex relationship influences the others, resulting in a chain-like structure for the regional sustainable development capacity evaluation system. After a detailed analysis was carried out on the index system, some indexes such as the rapid development of the economy and social construction ability were found to effectively reflect the regional carrying capacity and the production capacity of the complex regional system. As these indexes can effectively promote regional sustainable development capacity, they can be classified as efficiency indexes. Indexes such as waste emissions and birth rate belong to pressure indexes, which can have a certain negative impact on regional sustainable development. Combining the concepts of information entropy and dissipative structure theory, the regional system comprised of the economic, social and environmental subsystems can be redefined, with positive and negative entropy flows and change as the key points, so as to study the sustainable development condition of each subsystem. In view of this, based on the principles of scientific nature and operability, comprehensiveness and representativeness, we chose China’s Sichuan Province from 2007–2015 as the basis to build the regional sustainable development ability index system, as shown in Table 1.

**Figure 1:**
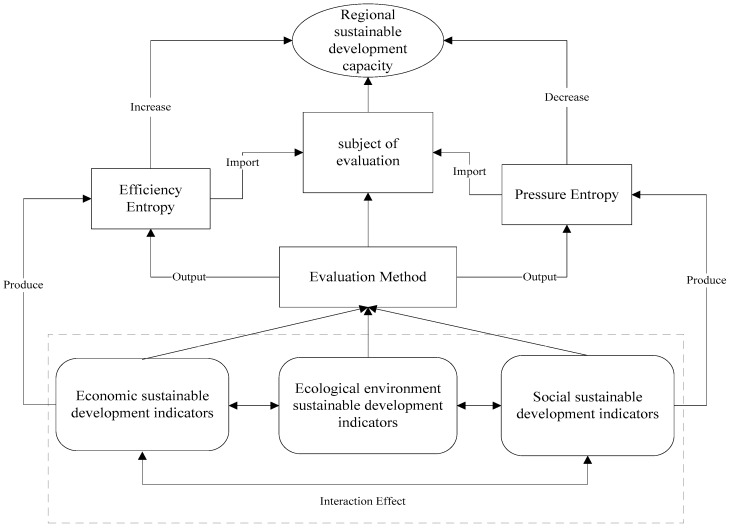
Index support figure.

In this paper, the economic, urban ecological environment and social sustainable development capacity were chosen as first grade indexes from the literature review and the Sichuan Province 2008–2016 statistical yearbook. Then, combined with entropy change, these first-grade indexes were divided into positive efficiency indexes and negative pressure indexes, so a more comprehensive index system was established. The indexes are not completely independent each other. To some extent, urban economic development promotes the city’s social and environmental construction, and the environmental index influences development and promotes the development of the urban economy and society, and a harmonious social atmosphere affects economic development and environmental protection. Urban sustainable development capacity indexes are closely related to each other, and provide decision support for urban sustainable development capacity measurements (as shown in Figure 1). For urban sustainable development capacity analysis, the relationships between the three first grade indexes and their independence need to be considered.

## 3. Measurement Methods for Regional Sustainable Development Capacity

### 3.1. Measurement Based on the Information Entropy-Brusselator Model

The information entropy concept was first proposed by Shannon in 1948 as an application concept in information theory. As entropy theory developed, the information entropy concept was gradually introduced into many fields to allow for the exploration of the orderly degree of a system. Information entropy can effectively describe the order degree of an organized system and highlight the disorganized degree in the quantitative performance of a system. The greater the degree of disorganization, the lower the degree of order. On the contrary, the smaller the information entropy, the smaller the degree of disorganization, and the higher the degree of order. Information entropy theory can be used for all statistical process analysis and has the advantage of overcoming the problems of complexity and uncertainty [22]. In this paper, regional sustainable development was regarded as the complex system, and information entropy was introduced to analyze the changes in regional entropy trends to measure the regional sustainable development capacity. Research on sustainable development capacity can be conducted by judging whether the system is a dissipative structure.

**Figure 2:**
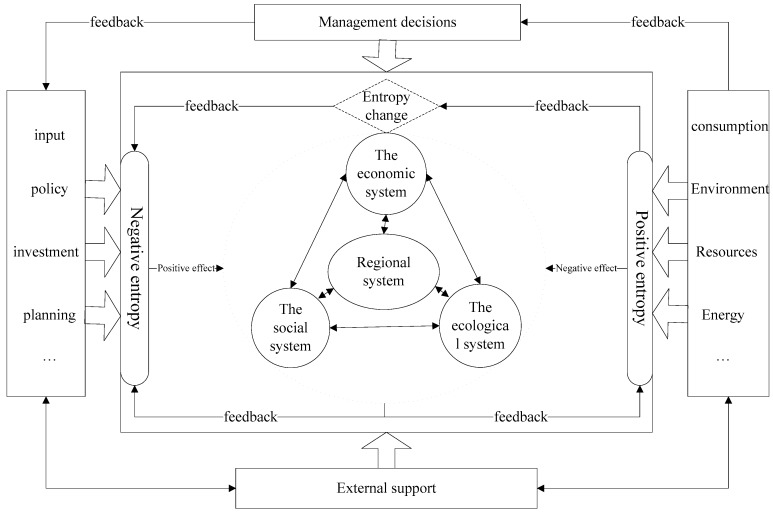
The framework for regional system development.

From the analysis of the regional system, it was found that along with energy production and exchange, regional internal operations and the external environment have close material and information transformations. At this level, the regional system matches the assumption of a dissipative structure. Therefore, the regional system quantitative method using information entropy is feasible for this paper. The regional system operation needs inner workings (human activities) to achieve material, energy and information production and exchange, so the system is open all the time. According to dissipative structure theory, information entropy flows in regional systems can be divided into efficiency entropy flows and pressure entropy flows according to their properties [23]. Pressure entropy flow (positive entropy) is the root of chaos in a regional system, as it encompasses human activities, increasing birth rates, pollutant emissions and energy consumption, all of which could have a negative influence on the regional system. Negative influences result in an increase in pressure entropy flow (positive entropy), which is closely related to spontaneity and initiatives in the system. Contrary to the positive entropy, the efficiency entropy flow (negative entropy) can effectively decrease the influence of positive entropy and eliminate the regional system’s internal chaos. In an open system, government construction investment, environmental protection and social construction can increase the negative entropy flow, meaning that the regional system constantly exchanges energy and information with the outside environment. Such an exchange pushes the system to develop in an orderly direction and, therefore, increase the sustainable development capacity of the system as shown in Figure 2. The generation of negative entropy is because of the external environment and organization. Therefore, based on the theory of information entropy, whether an operating system has a higher regional sustainable development capacity can be judged from the change in the system’s internal entropy flow.

In a regional system, the efficiency entropy flow and the pressure entropy flow are independent. Whether the regional system has a dissipative structure requires an independent analysis of the internal system entropy flow, so the current state of the regional system is defined according to the changes in entropy value [24]. Therefore, the Brusselator model was introduced to study the relationship between entropy flows in the system and to identify whether the system had a dissipative structure [25,26,27].

$H^{+}$ and $H^{-}$ can be set as the positive entropy flow and negative entropy flow, respectively. Then, $h_{+}$ and $h_{-}$ represent their corresponding quantification factors. $S_{y}$ represents the dissipation structure and $S_{n}$ represents the dissipative structure, which are the possible states of the emergency supply chain system under the impact of positive and negative entropy flows [28,29,30]. According to the three molecular expressions in the Brusselator model, the management entropy for the emergency supply chain system can be expressed as:

(1) $$H^{+}\overset{k_{1}}{\to }h_{+}$$

(2) $$H^{-}+h_{+}\overset{k_{2}}{\to }h_{-}+S_{n}$$

(3) $$h_{-}+2h_{+}\overset{k_{3}}{\to }3h_{+}$$

(4) $$h_{+}\overset{k_{4}}{\to }S_{y}$$

where $k_{1}$–$k_{3}$ are constants, $h_{+}$ is the positive management entropy flow, and $h_{-}$ is the negative management entropy flow. Equations (1) and (3) promote an increase in $h_{+}$, and Equations (2) and (4) reduce $h_{+}$. Equation (2) helps increase the negative management entropy flow $h_{-}$, and Equation (4) results in its decrease.

Combined with the Brusselator principle, the equation below was established to study the entropy flow changes in regional sustainable development capacity:

(5) $$\left|H^{-}\right|-\left|H^{+}\right|\{\begin{array}{c} >0\;positive\;indexes\\ =0\;critical\;state\\ <0\;negative\;indexes \end{array}$$

**Figure 3:**
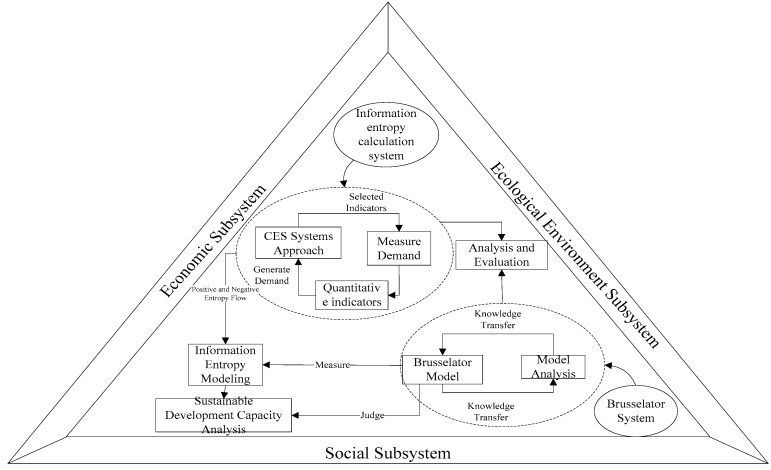
Information entropy-brusselator measurement model.

To learn how the information entropy-Brusselator model works, a working draft was built, as shown in Figure 3.

### 3.2. Calculating the Regional System’s Regional Information Entropy

The steps for the regional sustainable development capacity measurement, and to judge whether it is a dissipative structure are shown below. Data preprocessing for data standardization was needed before the calculation. Normalized processing was used for the data standardization in this paper. As the pressure entropy index and the efficiency entropy index need independent operations, their positives and negatives were ignored. The processing equation is as follows:

(6) $$X_{i}=\frac{x}{maxx}$$

The data after processing allows for the calculation index entropy changes throughout the years to be determined. For example, in the evaluation of the n index in m year, the information entropy of *i* index can be obtained using the following equation:

(7) $$h_{i}=-\frac{1}{lnm}\overset{m}{\underset{j=1}{\sum }}\frac{x_{ij}}{x_{i}}ln\frac{x_{ij}}{x_{i}}$$

where, $h_{i}$ is the information entropy of the i*_th_* index, and $x_{ij}$ is the standardized data for the i*_th_* index in year *j:* $x_{i}=\sum_{j=1}^{m}x_{ij}$

A quantitative measurement model for regional sustainable development capacity is required for the index information entropy calculation, so that the actual sustainable development capacity level of the regional system can be identified through the information entropy changes. Using the entropy weight method, the equation to calculate the weight of the *i_th_* index is:

(8) $$W_{i}=\left(1-h_{i}\right)/(n-\sum_{i=1}^{n}h_{i})$$

where $0\leq W_{i}\leq 1$, $\sum_{i=1}^{n}W_{i}=1$.

Superiority quantitative evaluation score values over the years based on information entropy can be determined by Equation (9):

(9) $$Q_{j}=\sum W_{i}X_{i}$$

where $W_{i}$ is index weight for each index and $X_{i}$ is the index standardized value of each index.

The results from Equation (9) can be further used to identify the change in each subsystem’s evaluation values. The equation for this is:

(10) $$△Q_{j}=\left|Q_{j}^{-}\right|-\left|Q_{j}^{+}\right|$$

where $Q_{j}^{+}$ is the pressure evaluation, and $Q_{j}^{-}$ is the efficiency evaluation.

Finally, the total entropy change in Sichuan Province system can be determined. According to the calculation results and dissipative structure theory, the system sustainable development capacity evaluation value for Sichuan Province can be expressed as the sum of the variance of the three subsystem evaluations. This expression is shown in Equation (11):

(11) $$△Q=△Q_{C}+△Q_{E}+△Q_{S}$$

**Figure 4:**
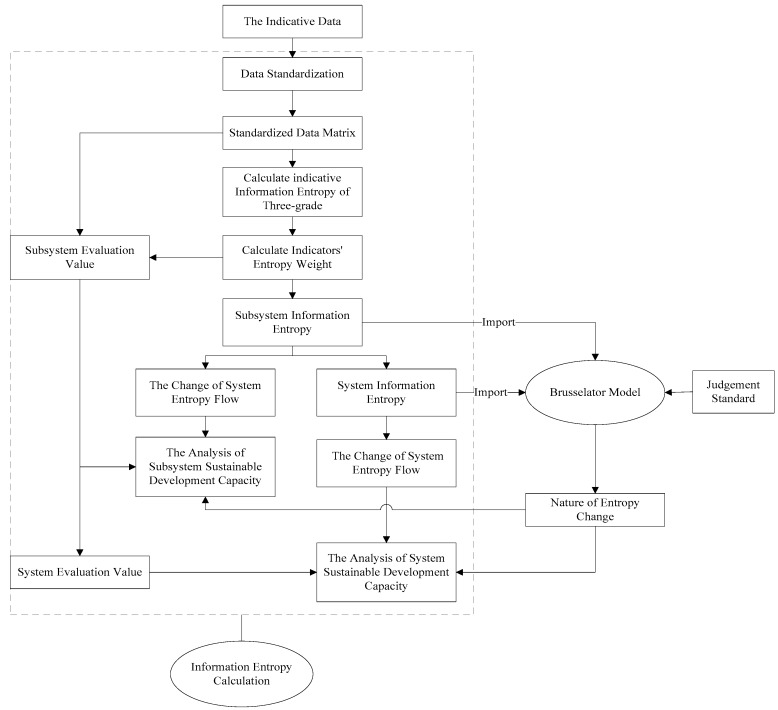
Technical flow chart.

The detailed process for the regional sustainable development measurement is shown in Figure 4.

## 4. Case Analysis

### 4.1. Data Sources

The data in the Sichuan Province 2008–2016 statistical yearbooks was analyzed to obtain the regional sustainable development evaluation data, as shown in Table 2.

### 4.2. Data Processing

The main purpose of this study was to analyze the sustainable development capacity of Sichuan Province. Information entropy in regional system development is needed. Therefore, the data in Table 2 need to be investigated the changing trends in the positive or negative entropy flow by independent mathematical analyses. Each index has different units that need to be dealt with before data standardization. According to the efficiency of the entropy flow and pressure entropy flows, the indexes each year are normalized and standardized results obtained. MATLAB (The Mathworks, Inc., Natick, MA, USA) was used to further process the data according to entropy weight theory. The index weights and the information entropy for each index are shown in Table 3:

The standardized data can be dealt with using Equations (7)–(11) to determine the efficiency entropy flow, the pressure entropy flow changes in each subsystem and the quantitative evaluation value over several years. The results are shown in Table 4 and Table 5.

### 4.3. Results and Discussion

To better observe each subsystem’s entropy flow over the years, the data from Table 4 and Table 5 was used to generate an entropy flow variation graph and a system value variation graph, as shown in Figure 5, Figure 6, Figure 7, Figure 8, Figure 9, Figure 10, Figure 11 and Figure 12.

**Figure 5:**
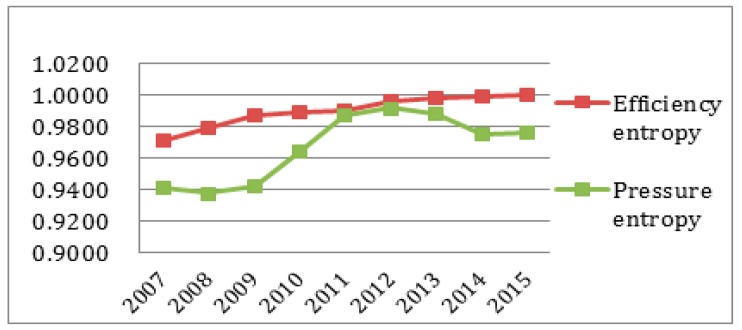
Entropy flow change in the economic subsystem.

**Figure 6:**
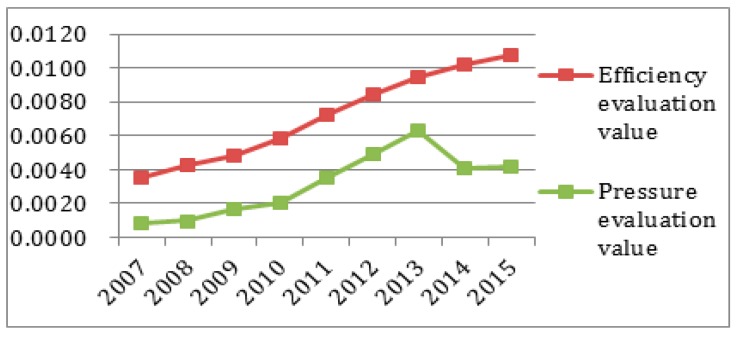
Evaluation value change in the economic subsystem.

It can be seen that in Figure 5 the efficiency entropy change is relatively stable over the years, suggesting that the economic subsystem support for the sustainable development capacity of the regional system in Sichuan Province is rising steadily. The pressure entropy flow has a sharp increase in 2010, and then is gradually increasing from 2011 to 2013, suggesting the sustainable development capacity is reducing. It declines in 2014, and keeps steady until 2015, suggesting that the economic sustainable development capacity of the subsystem is on the rise. Combined with Figure 5, it is easy to see that the efficiency entropy flow is always greater than the pressure entropy flow in the economic subsystem. From the perspective of the value analysis (Figure 6), although the pressure evaluation value reaches the highest value in 2013, but the efficiency value does not catch up, and the efficiency value is always growing faster than the pressure value and the sustainable development of the economic subsystem capacity keeps increasing.

**Figure 7:**
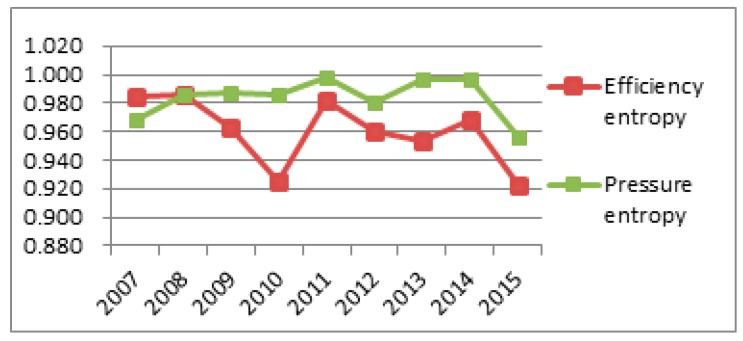
Entropy flow change in the ecological subsystem.

**Figure 8:**
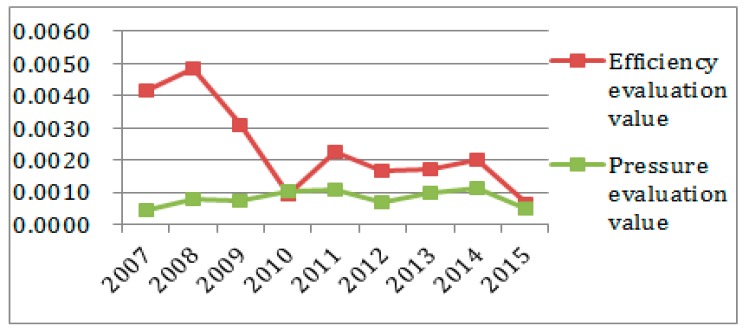
Evaluation value change in the ecological subsystem.

In Figure 7, the ecological environmental subsystem efficiency entropy flow is always lower, and has almost the same growth trend as the pressure entropy flow, except for 2007, which shows that the matters concerning pollutant emissions, energy consumption and nature environmental protection are increasingly serious. The efficiency entropy flow is higher than the pressure entropy flow in 2007, which proves that ecological environment problems have not been paid due attention. Meanwhile, as shown in Figure 8, the efficiency evaluation values is declining sharply in 2009, and the efficiency evaluation values are seriously below the pressure evaluation values after 2009. Combined with the entropy change and the evaluation value in the ecological environmental subsystem in Figure 11, the entropy change is negative, indicating that although measures are being taken in a timely manner, the effect is not so good. The sustainable development capacity in the ecological environmental subsystem is weak and needs effective measures to promote the sustainable development capacity.

**Figure 9:**
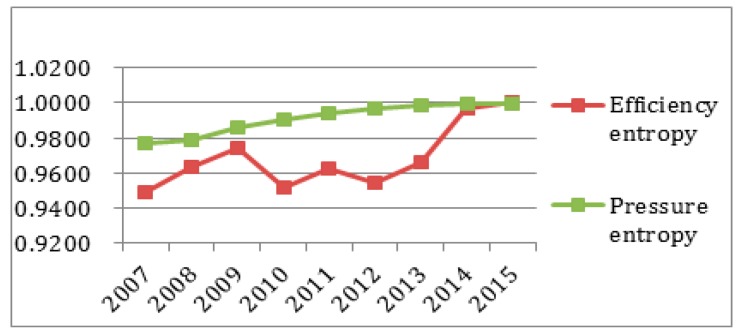
Entropy flow change in the social subsystem.

Figure 9 shows that the social efficiency entropy flow has a smooth growth between 2007 and 2009, and a sharp decrease in 2010, then, it remains steady from 2010 to 2013, while at the same time, the pressure entropy flow is rising steadily and higher than the efficiency entropy flow. However, the value of the efficiency entropy and the pressure entropy is the same from 2014 to 2015. This suggests that the social subsystem maintains an internal balanced development. In the light of the development trend, the social subsystem will support for the sustainable development capacity of the regional system in Sichuan Province.

**Figure 10:**
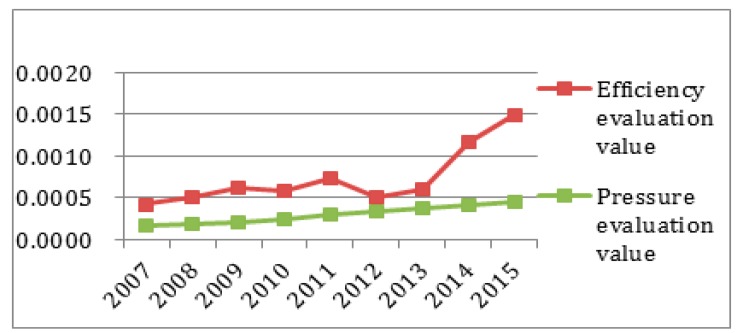
Evaluation value change in the social subsystem.

Combined with Figure 10, the pressure evaluation flow is lower than the efficiency evaluation flow over the past years, so Sichuan in general needs to invest more in social development, people's livelihood security and development mode transformation to improve social equality and promote the sustainable development capacity of the social subsystem.

**Figure 11:**
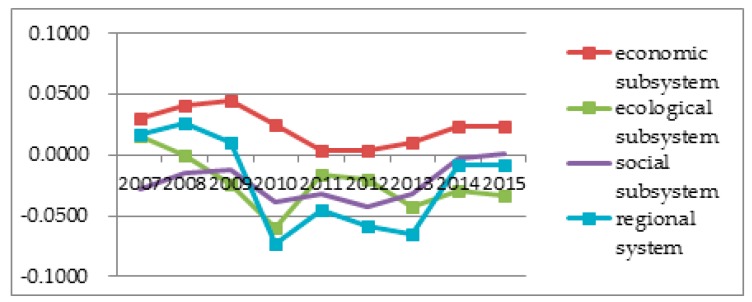
Entropy flow change in the regional sustainable development capacity.

In Figure 11, the ecological environment subsystem entropy is negative, except in 2007, and the social subsystem entropy is also always negative, indicating there are no effective measures in Sichuan Province to promote ecological environment and social sustainable development. Although Sichuan Province’s economy has experienced rapid development, the investment ratio in social development and people’s livelihood security declines, which results in slower ecological environment and social development than economic development.

**Figure 12:**
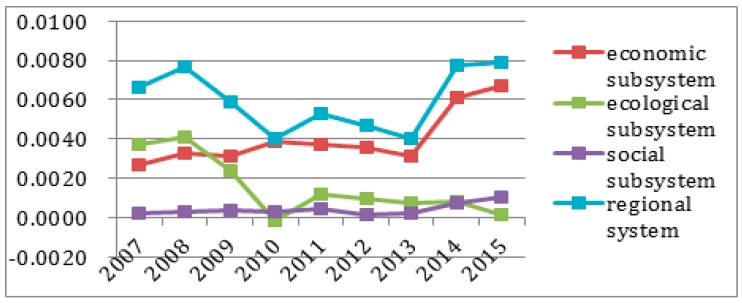
Evaluation value change in the regional sustainable development capacity.

It can be observed in Figure 12 that the sustainable development capacity decreases over the years. Before 2008, the sustainable development capacity of Sichuan Province showed a rising trend, which shows it had a certain sustainable development ability. However, after 2008, the regional sustainable development capacity flow has a big decline until to 2013. After 2013, the sustainable development capacity shows an increase, the effect is very obvious. After the analysis of the three subsystems’ entropy value changes and evaluation values, it can be found that the changes in Sichuan’s sustainable development capacity and ecological environmental subsystem are very similar (Figure 12).

Although the rapid development of the economy plays an important role in enhancing regional sustainable development, it cannot offset the impact of the ecological environmental subsystem or the social subsystem for the sustainable development capacity in Sichuan Province. The sustainable development of the ecological environmental subsystem and the social subsystem is also very important.

The above analysis of the three subsystems shows that Sichuan Province needs reasonable planning to promote the virtues of economic, environmental and social development. In view of the change of the entropy flow and the overall system in the three systems, the following proposals are given:

1. Economic strength is the power of regional sustainable development. Sichuan Province needs to have scientific planning to arrange the regional economic structure, promote the regional carrying capacity, and develop basic and potential industries.
2. Better ecological condition is a prerequisite for regional sustainable development. Cities should put more effort into green development, resource recycling, energy conservation, and emissions reductions. For example, by developing new energy industries, having a comprehensive utilization of resources and ecological resources, and maintaining a circular economy to promote the sustainable development of the regional ecological environment.
3. Regional harmony is the foundation of regional sustainable development. In the process of regional sustainable development, social security construction is basic. Increasing spending on social construction, strengthening social security and regional infrastructure, and smoothing regional development are very important in creating a good social order and living environment for area residents.
4. For a city’s sustainable development, the equal distribution of resources is also very important, so Sichuan Province should take some measures to allocate the resources more equally. Such as making more poor people who came from the ethnic areas to afford the cost of the doctor and have more children who came from the poor areas can receive good education. The increasing of social equality is helpful to improve the ability of the sustainable development of the social system, so as to improve the Sichuan Province sustainable development capacity.
5. In terms of the sustainable development of the whole system, there needs to be a comprehensive evaluation of the existing sustainable development level in Sichuan Province. Then corresponding laws and policies system should be developed which highlight the coordinated development of the economy, society and the ecological environment. In this way, economic development advantages could be made full use of to promote environmental protection and social construction. Finally, the sustainable utilization of resources, economic development, ecological environment development and social construction could make up a virtuous circle.

## 5. Conclusions

Using the 2008 to 2016 Sichuan Province statistical yearbook, this paper established an index system for regional sustainable development capacity to comprehensively analyze the economic subsystem, environmental subsystem and social subsystem in Sichuan Province. Combined with the use of the information entropy principle, an analysis of the entropy flow change was conducted to derive a comprehensive evaluation value of the regional sustainable development capacity. From this research, the following conclusions were made:

1. Based on information entropy theory, traditional qualitative analysis can be converted to vector calculations to avoid the influence of subjective preferences. Through the establishment of an information entropy-Brusselator judgment model, the economic, ecological environment and the social subsystem entropy value changes can be analyzed so as to identify the sustainable development capacity of each subsystem and the whole province.
2. The information entropy model established in this paper can be used to find the advantages and disadvantages in system operations and can therefore assist in finding a more reasonable internal improvement focus for the system to provide a decision basis for effective improvements.
3. Through the analysis of each subsystem entropy flow change in Sichuan Province, it was found that as the economy develops, the complexity of the economic subsystem, environmental subsystem and the social subsystem increase. Although the sustainable development ability in Sichuan Province is rising, its ecological environmental protection and social construction still need to be strengthened.

On the whole, the highlights of this paper can be summed up in three points. First of all, in order to conduct a comprehensive and objective analysis for the regional sustainable development capacity, the paper introduced forty-six indicators and established a relatively complete index system. Afterwards, the model which is built by information entropy and Brusselator can effectively reduce the confusion of the whole system on problems solving, and get a clear research results about the three subsystems internal change and the overall change of the regional system. Finally, this paper can be applied to the relevant study about the index system establishing of sustainable development or the evaluation of regional sustainable development capacity.

The established index system comprehensively considers various factors, but there are still certain limitations. The index system does not contain all the regional sustainable development capacity influencing factors. In addition, this paper focused on a static independent analysis of the data indexes, without studying the internal logic relationships between the indexes and a dynamic evaluation of the indexes. Therefore, further improvements to the evaluation index system are planned as a focus at a later time to further study the internal logical relationships and to dynamically evaluate the index.

## Figures and Tables

**Table 1:** Index system used to evaluate sustainable development.

Target-Grade	First-Grade Index	Second-Grade Index	Third-Grade Index
Regional sustainable development capacity	Regional economic sustainable development capacity C	Efficiency indexes C1	Gross Domestic Product C11
			Primary Industry-owned Value Added C12
			Secondary Industry-owned Value Added C13
			Tertiary Industry-owned Value AddedC14
			Agriculture, Forestry, Animal Husbandry and Fishery-owned Value Added C15
			Industry-owned Value Added C16
			Construction-owned Value Added C17
			Finance-owned Value Added C18
		Pressure indexes C2	Total Investment in Fixed Assets C21
			Total Investment in Fixed Assets in Agriculture, Forestry, Animal Husbandry and Fishery C22
			Total Investment in Fixed Assets in Industry C23
			Total Investment in Fixed Assets in Construction C24
			Total Investment in Fixed Assets in Finance C25
			Total Investment in Fixed Assets in Real Estate C28
	Regional environment sustainable development capacity E	Efficiency indexes E1	Complete investment industrial pollution control E11
			Investment in waste water treatment E12
			Investment in waste gas treatment E13
			Investment in waste solid treatment E14
			Total investment in fixed assets of energy industry E15
			Forest Area E16
			Forest Coverage Rate E17
			Area of Weltlands E18
			Green Covered are as % of Completed Area E19
		Pressure indexes E2	Total Emissions of SO2 E21
			Total Waste Water Discharged E22
			Total Emissions of Waste Oxygen Water Needed by Chemistry E23
			Total Emissions of Ammonia and Nitrogen E24
			Total Volume of Natural Gas E25
			Total Volume of Water Supply E26
			Total Volume of Electricity Consumption E27
			Geological Disasters E28
	Regional social sustainable development capacity S	Efficiency indexes S1	Education Expenditure S11
			Culture, Sport and Media Expenditure S12
			Social Safety Net and Employment Effort Expenditure S13
			Medical and Health Care Expenditure S14
			Number of Civil Vehicle S15
			Per Capita Area of Roads S16
			Number of Community Service Facilities S17
		Pressure indexes S2	Population Birth Rate S21
			Population Death Rate S22
			Population Natural Growth Rate S23
			Unemployed Persons in Urban Area S24
			Unemployment Rate in Urban Area S25
			Per Capita Consumption of Residents S26

**Table 2:** Index data.

C	C1	**Index**	**Unit**	**2007**	**2008**	**2009**	**…**	**2013**	**2014**	**2015**
		C11	$\times 10^{8}\;\mathrm{RMB}$	10,562.39	12,601.23	14,151.28	…	26,392.07	28,536.66	30,053.10
		C12	$\times 10^{8}\;\mathrm{RMB}$	2032	2216.15	2240.61	…	3368.66	3531.05	3677.30
		C13	$\times 10^{8}\;\mathrm{RMB}$	4648.79	5823.39	6711.87	…	13,472.05	13,962.41	13,248.08
		C14	$\times 10^{8}\;\mathrm{RMB}$	3881.6	4561.69	5198.8	…	9551.36	11,043.2	13,127.72
		C15	$\times 10^{8}\;\mathrm{RMB}$	2032	2216.15	2240.61	…	3425.61	3594.17	3745.32
		C16	$\times 10^{8}\;\mathrm{RMB}$	3921.41	4956.13	5678.24	…	11,540.86	11,851.99	11,039.08
		C17	$\times 10^{8}\;\mathrm{RMB}$	727.38	867.26	1033.63	…	2038.17	2225.44	2321.38
		C18	$\times 10^{8}\;\mathrm{RMB}$	335.13	433.82	561.50	…	1755.22	1953.51	2202.23
	C2	C21	$\times 10^{8}\;\mathrm{RMB}$	5639.8	7127.81	11,371.87	…	21,049.15	23,577.17	25,973.74
		C22	$\times 10^{8}\;\mathrm{RMB}$	176.18	268.44	441.4	…	667.23	752.16	1001.56
		C23	$\times 10^{8}\;\mathrm{RMB}$	1455.6	2018.62	3016.92	…	4940.71	5020.76	5193.37
		C24	$\times 10^{8}\;\mathrm{RMB}$	36.51	40.92	75.91	…	52.46	47.71	101.01
		C25	$\times 10^{8}\;\mathrm{RMB}$	3.6	3.49	7.79	…	62.8	27.67	22.98
		C26	$\times 10^{8}\;\mathrm{RMB}$	1573	1791.02	2749.42	…	6214.99	7193.74	7757.92
E	E1	E11	$\times 10^{4}\;\mathrm{RMB}$	201,033	193,808	96,191	…	188,392	232,452	118,258
		E12	$\times 10^{8}\;\mathrm{RMB}$	9.9757	7.5227	5.2686	…	2.9791	5.4168	5.5112
		E13	$\times 10^{4}\;\mathrm{RMB}$	83,307	86,503	31,900	…	148,926	164,883	46,883
		E14	$\times 10^{4}\;\mathrm{RMB}$	8368	10,196	6840	…	1407	1689	250
		E15	$\times 10^{8}\;\mathrm{RMB}$	654.5	677.3	823.8	…	1574.3	1429.3	1603.0
		E16	$\times 10^{4}\;m^{2}$	1464.34	1464.34	1703.74	…	1703.74	1738.16	1870.66
		E17	%	30.3	30.3	35.2	…	35.2	35.76	38.65
		E18	$\times 10^{8}\;m^{2}$	96.17	96.17	96.17	…	174.78	174.78	174.78
		E19	%	34.2	35.3	36.4	…	38.4	37.51	38.7
	E2	E21	$\times 10^{4}\;t$	117.87	114.8	113.53	…	81.67	79.64	71.65
		E22	$\times 10^{8}\;t$	25.2961	26.2343	26.2709	…	30.7648	33.1277	34.1607
		E23	$\times 10^{4}\;t$	77.1	74.9	74.8	…	123.2	121.63	118.64
		E24	$\times 10^{4}\;t$	5.97	6.19	5.95	…	13.7	13.47	13.14
		E25	$\times 10^{8}\;m^{3}$	62.99	59.5	51.11	…	59.02	61.01	62.98
		E26	$\times 10^{8}\;m^{3}$	213.98	207.64	223.46	…	242.47	236.9	265.5
		E27	$\times 10^{8}$ KW/h	1177.51	1210.13	1324.61	…	1948.95	2014.79	1992.40
		E28	unit	934	2161	1997	…	2161	2758	349
S	S1	S11	$\times 10^{8}\;\mathrm{RMB}$	451.44	540.65	684.66	…	540.65	1056.9	1252.33
		S12	$\times 10^{8}\;\mathrm{RMB}$	45.7	59.37	87.35	…	59.37	135.64	139.41
		S13	$\times 10^{8}\;\mathrm{RMB}$	422.36	513.65	645.79	…	513.65	927.01	1111.75
		S14	$\times 10^{8}\;\mathrm{RMB}$	219.1	263.34	372.96	…	263.34	584.1	686.4
		S15	$\times 10^{8}$ unit	183.62	219.05	284.69	…	573.03	666.92	767.13
		S16	sq. m	11.5	11.84	12.14	…	11.84	13.32	13.63
		S17	unit	4872	5559	5768	…	5552	12,790	18,762
	S2	S21	‰	9.21	9.54	9.15	…	9.9	10.22	10.30
		S22	‰	6.29	7.15	6.43	…	6.9	7.02	6.94
		S23	‰	2.92	2.39	2.72	…	3	3.2	3.36
		S24	$\times 10^{4}\;\mathrm{person}$	34.53	37.86	36.28	…	42.87	54.36	54.64
		S25	%	4.2	4.6	4.3	…	4.1	4.2	4.1
		S26	RMB	5259	6072	6863	…	12,485	13,755	14,774.00

**Table 3:** Data standardization and processed results for information entropy.

C		**Index**	**2007**	**2008**	**2009**	**...**	**2013**	**2014**	**2015**	**Entropy**	**Weight**
	C1	C11	0.351	0.419	0.471	…	0.878	0.950	1.000	0.974	0.019
		C12	0.553	0.603	0.609	…	0.916	0.960	1.000	0.990	0.004
		C13	0.333	0.417	0.481	…	0.965	1.000	0.949	0.972	0.008
		C14	0.296	0.347	0.396	…	0.728	0.841	1.000	0.967	0.008
		C15	0.543	0.592	0.598	…	0.915	0.960	1.000	0.989	0.002
		C16	0.331	0.418	0.479	…	0.974	1.000	0.931	0.972	0.005
		C17	0.313	0.374	0.445	…	0.878	0.959	1.000	0.968	0.004
		C18	0.152	0.197	0.255	…	0.797	0.887	1.000	0.922	0.010
	C2	C21	0.217	0.274	0.438	…	0.810	0.908	1.000	0.956	0.005
		C22	0.176	0.268	0.441	…	0.666	0.751	1.000	0.950	0.005
		C23	0.280	0.389	0.581	…	0.951	0.967	1.000	0.971	0.003
		C24	0.361	0.405	0.752	…	0.519	0.472	1.000	0.978	0.002
		C25	0.057	0.056	0.124	…	1.000	0.441	0.366	0.851	0.011
		C26	0.203	0.231	0.354	…	0.801	0.927	1.000	0.943	0.004
E	E1	E11	0.865	0.834	0.414	…	0.810	1.000	0.509	0.972	0.002
		E12	1.000	0.754	0.528	…	0.299	0.543	0.552	0.975	0.002
		E13	0.505	0.525	0.193	…	0.903	1.000	0.284	0.930	0.004
		E14	0.821	1.000	0.671	…	0.138	0.166	0.025	0.847	0.009
		E15	0.408	0.423	0.514	…	0.982	0.892	1.000	0.978	0.001
		E16	0.783	0.783	0.911	…	0.911	0.929	1.000	0.999	0.000
		E17	0.784	0.784	0.911	…	0.911	0.925	1.000	0.999	0.000
		E18	0.550	0.550	0.550	…	1.000	1.000	1.000	0.980	0.001
		E19	0.884	0.912	0.941	…	0.992	0.969	1.000	1.000	0.000
	E2	E21	1.000	0.974	0.963	…	0.693	0.676	0.608	0.993	0.000
		E22	0.741	0.768	0.769	…	0.901	0.970	1.000	0.997	0.000
		E23	0.592	0.575	0.574	…	0.946	0.934	0.911	0.987	0.001
		E24	0.415	0.431	0.414	…	0.953	0.937	0.914	0.967	0.001	
		E25	1.000	0.945	0.811	…	0.937	0.969	1.000	0.999	0.000	
		E26	0.806	0.782	0.842	…	0.913	0.892	1.000	0.999	0.000	
		E27	0.584	0.601	0.657	…	0.967	1.000	0.989	0.991	0.000	
		E28	0.297	0.686	0.634	…	0.686	0.876	0.111	0.941	0.002	
S	S1	S11	0.360	0.432	0.547	…	0.432	0.844	1.000	0.969	0.001	
		S12	0.321	0.417	0.613	…	0.417	0.953	0.979	0.959	0.001	
		S13	0.380	0.462	0.581	…	0.462	0.834	1.000	0.975	0.001	
		S14	0.319	0.384	0.543	…	0.384	0.851	1.000	0.964	0.001	
		S15	0.239	0.286	0.371	…	0.747	0.869	1.000	0.957	0.001	
		S16	0.844	0.869	0.891	…	0.869	0.977	1.000	0.999	0.000	
		S17	0.260	0.296	0.307	…	0.296	0.682	1.000	0.912	0.002	
	S2	S21	0.894	0.926	0.888	…	0.961	0.992	1.000	0.999	0.000	
		S22	0.880	1.000	0.899	…	0.965	0.982	0.971	1.000	0.000	
		S23	0.869	0.711	0.810	…	0.893	0.952	1.000	0.997	0.000	
		S24	0.632	0.693	0.664	…	0.785	0.995	1.000	0.993	0.000	
		S25	0.913	1.000	0.935	…	0.891	0.913	0.891	1.000	0.000	
		S26	0.356	0.411	0.465	…	0.845	0.931	1.000	0.974	0.001	

**Table 4:** System entropy flow change.

Item		Year						
		2007	2008	2009	…	2013	2014	2015
Economic subsystem	Efficiency entropy flow $h_{c}^{-}$	0.971	0.979	0.987	…	0.998	0.999	1.000
	Pressure entropy flow $h_{c}^{+}$	0.941	0.937	0.942	…	0.988	0.975	0.976
	Entropy change $△\;h_{C}$	0.030	0.042	0.045	…	0.010	0.024	0.024
Ecological environment subsystem	Efficiency entropy flow $h_{E}^{-}$	0.984	0.985	0.963	…	0.954	0.968	0.923
	Pressure entropy flow $h_{E}^{+}$	0.969	0.986	0.987	…	0.996	0.997	0.956
	Entropy change $△\;h_{E}$	0.015	−0.001	−0.024	…	−0.042	−0.029	−0.033
Social subsystem	Efficiency entropy flow $h_{S}^{-}$	0.949	0.963	0.975	…	0.966	0.997	1.000
	Pressure entropy flow $h_{S}^{+}$	0.977	0.978	0.986	…	0.999	1.000	1.000
	Entropy change $△\;h_{S}$	−0.028	−0.015	−0.011	…	−0.033	−0.003	0.000
Total entropy flow change △ h		0.017	0.026	0.010	…	−0.065	−0.008	−0.009

**Table 5:** Quantitative evaluation of the system.

Item		Year						
		2007	2008	2009	…	2013	2014	2015
Economic subsystem	Efficiency evaluation value $Q_{c}^{-}$	0.003	0.004	0.005	…	0.009	0.010	0.011
	Pressure evaluation value $Q_{c}^{+}$	0.001	0.001	0.002	…	0.006	0.004	0.004
	variation value $△\;Q_{C}$	0.002	0.003	0.003	…	0.003	0.006	0.007
Ecological environment subsystem	Efficiency evaluation value $Q_{E}^{-}$	0.004	0.005	0.003	…	0.002	0.002	0.001
	Pressure evaluation value $Q_{E}^{+}$	0.000	0.001	0.001	…	0.001	0.001	0.001
	variation value $△\;Q_{E}$	0.004	0.004	0.002	…	0.001	0.001	0.000
Social subsystem	Efficiency evaluation value $Q_{S}^{-}$	0.000	0.001	0.001	…	0.001	0.001	0.001
	Pressure evaluation value $Q_{S}^{+}$	0.000	0.000	0.000	…	0.000	0.000	0.000
	variation value $△\;Q_{S}$	0.000	0.001	0.001	…	0.001	0.001	0.001
Total evaluation value change △ Q		0.006	0.008	0.006	…	0.005	0.008	0.008
